# The Polygenic Nature of Multiple Sclerosis: Genetic Variants, Immunological Modulation, and Environmental Connections

**DOI:** 10.2174/0118715303325979241206115417

**Published:** 2024-12-31

**Authors:** Zuber Khan, Sidharth Mehan, Pankaj Kumar Maurya, Aakash Kumar, Ghanshyam Das Gupta, Acharan S. Narula, Reni Kalfin

**Affiliations:** 1 Division of Neuroscience, Department of Pharmacology, ISF College of Pharmacy, Moga, Punjab, India; Affiliated to IK Gujral Punjab Technical University, Jalandhar, Punjab, 144603, India;; 2 Department of Pharmaceutics, ISF College of Pharmacy, Moga, Punjab, India Affiliated to IK Gujral Punjab Technical University, Jalandhar, Punjab, 144603, India;; 3 Narula Research, LLC, 107 Boulder Bluff, Chapel Hill, NC 27516, USA;; 4 Institute of Neurobiology, Bulgarian Academy of Sciences, Acad. G. Bonchev St., Block 23, Sofia, 1113, Bulgaria;; 5 Department of Healthcare, South-West University “NeofitRilski”, Ivan Mihailov St. 66, Blagoevgrad, 2700, Bulgaria

**Keywords:** Multiple sclerosis, genetic variants, genome-wide association study, demyelination, polygenic risk score, diagnosis

## Abstract

Multiple Sclerosis (MS), a debilitating inflammatory disorder of the central nervous system characterized by demyelination, is significantly influenced by polygenic variations. Although the precise cause of MS remains unclear, it is believed to arise from a complex interplay of genetic and environmental factors. Recent investigations have focused on the polygenic nature of genetic alterations linked to MS risk. This review highlights the critical role of these genetic variants in shaping disease susceptibility and progression. Specific Human Leukocyte Antigen (HLA) alleles, such as HLA-DRB1*15:01, HLA-DRB50*101, HLA-DR2+, HLA-DQ6, DQA 0102, and DQB1 0602, are implicated in immune modulation, significantly increasing the risk of developing MS. Additionally, Genome-wide Association Studies (GWAS) have identified non-HLA genetic variants that contribute to MS susceptibility, including IL-2RA (rs2104286), IL-7R (rs6897932), CD40 (rs1883832 T), CD58 (rs2300747), and others, each playing a role in immune regulation and disease progression. Dysfunctions in genes regulating myelin integrity, such as MOG (Myelin Oligodendrocyte Glycoprotein), MAG (Myelin-associated Glycoprotein), and PLP1 (Proteolipid Protein 1), further drive MS pathogenesis. Moreover, viral infections, notably Epstein-Barr Virus (EBV), Human Herpesvirus 6 (HHV-6), and measles virus, may exacerbate the development of MS by triggering immune responses. Understanding the contribution of these genetic and viral factors may shed light on the complex etiology of MS. Polygenic Risk Scores (PRS) provide a valuable tool for estimating MS susceptibility based on the cumulative effect of genetic variants. However, translating these genetic insights into clinical practice requires further validation, including environmental considerations. Investigating MS polygenicity could lead to personalized therapies, enhancing diagnosis, prognosis, and treatment, ultimately improving outcomes for MS patients.

## 1 INTRODUCTION

Multiple Sclerosis (MS) is classified as an autoimmune disorder predominantly impacting the Central Nervous System (CNS), which includes the brain and spinal cord [1]. MS is characterized by an autoimmune response where the immune system targets and damages myelin, the protective sheath around nerve fibers. This immune-mediated process leads to inflammation and the subsequent loss of myelin in individuals with MS, affecting the regular transmission of electrical impulses within neurons. The severity of the disease varies based on factors, such as age and the duration of the illness [2, 3]. The McDonald criteria, proposed by Thompson *et al.* in 2018 and previously outlined by Compston and Coles in 2008, use Magnetic Resonance Imaging (MRI) findings of white matter lesions in both the brain and spinal cord, the presence of Oligoclonal Bands (OCB) in the cerebrospinal fluid, and the manifestation of physical symptoms as diagnostic indicators [4, 5]. MS is a multifaceted disease characterized by elusive etiology, influenced by genetic and environmental factors [6, 7]. MS can manifest at any stage of life, but it is observed more frequently in females, occurring three times more often than in males. Typically, the onset of MS is observed between the ages of 20 and 40, as noted by Khan *et al.* and Dimitrov and Turner [1, 8, 9].

The concept of a polygenic nature pertains to the involvement of multiple genes in the development and progression of a disease. MS is widely recognized as a polygenic disorder, indicating that it arises from the collective influence of numerous gene variants, known as polymorphic genes, rather than a single gene [10]. Researchers have utilized GWAS to identify genetic variations associated with MS. These studies have involved a comparative analysis of genomic profiles of individuals diagnosed with MS and those without neurological disorders, aiming to detect genetic variations more prevalent in the MS cohort [11]. Numerous investigations have identified a substantial number of genetic variations associated with MS, a significant proportion of which are implicated in immune system processes [11-14]. Despite GWAS's efficacy in identifying trait-related variants, challenges, such as population stratification and significant polygenicity, persist. Additionally, GWAS presents ethical considerations that require a thorough examination [14, 15].

Although no definitive genetic factor responsible for MS has been established, substantial evidence supports the notion of inherited susceptibility to the disease [16, 17]. The presence of a first-degree relative, such as a parent or sibling, with the disease is associated with an elevated risk of developing MS. However, the overall risk remains relatively low [18, 19]. The research study conducted by Sawcer *et al.* has demonstrated the importance of genetic factors in disease development, as evidenced by studies involving twins and familial clustering [20]. The study by Lee *et al.* found a higher level of clinical concordance in monozygotic or identical twins compared to dizygotic or fraternal twins [21], suggesting a potential contribution to the condition's decreased prevalence. Recent studies by Sharma *et al.* and Goris *et al.* indicate that siblings of MS patients have a significantly higher risk of developing the disease, with a 7-fold increase compared to the general population [22, 23].

Additionally, approximately 15% of patients report a family member suffering from the illness. A genomic cluster of genes within the HLA region on chromosome 6 correlates with increased susceptibility to MS. This susceptibility is associated with the presence of specific genetic markers, including HLA-DR2 positivity, the DQA 0102 and DQB1 0602 alleles, and the HLA-DRB1, DR15, DRB1*1501, and DRB1*1503 genes [24-28]. These genes regulate the immune system and suggest that immune response abnormalities may contribute to MS development [29].

A recent study in the United States revealed a definitive correlation between the risk allele rs10191329 of the DYSF-ZNF638 locus and a shorter median duration before individuals who are homozygous carriers require walking assistance. This variant is also associated with more severe brainstem and cortical pathology in brain tissue. Significant genetic enrichment in CNS tissues also identified a potential association between rs149097173 in the DNM3-PIGC gene and this phenomenon [30]. Certain ethnic groups, particularly those of Northern European descent, show a heightened susceptibility to disease progression. Amezcua and Mccauley suggested that distinct genetic traits in different populations significantly impact MS pathogenesis [31]. Other significant genes in the MS context include IL-7 and IL-2 receptor alpha. In India, the protective allele for MS is HLA-DRB1*14:04, whereas the predisposing alleles are HLA-DRB1*15:01, 15:02, and DQB1*06:02 [32].

It is hypothesized that environmental exposure and a genetic predisposition contribute to MS development, showing variability among individuals [33]. MS is more prevalent in regions far from the equator, suggesting an association of sun exposure and vitamin D insufficiency with MS progression. Sunlight exposure enhances vitamin D production in the human body, which has immune-modulatory properties. Sintzel *et al.* and Breuer *et al.* indicated that inadequate sunshine exposure or diet might lead to low vitamin D levels, increasing MS susceptibility [34, 35]. Several viral infections, including Epstein-Barr Virus (EBV), Human Herpesvirus 6 (HHV-6), measles virus, and various retroviruses, have been linked to increased susceptibility to illness progression [36, 37].

However, the exact correlation between these viral infections and MS is under investigation. There is evidence of a correlation between tobacco smoking and an increased susceptibility to MS, as well as a worsening in the disease's progression and severity [38-40]. Other factors, such as toxicity, hormonal disorders, obesity, and stress, have been examined, but currently lack conclusive evidence [41-43].

Mazdeh *et al.* investigated the associations among a specific single nucleotide polymorphism (SNP), rs10735781, and other haplotypes within the EVI5 gene in relation to the vulnerability to Relapsing-remitting MS (RRMS) in the Iranian population [44]. Additionally, the study by Johnson *et al.* provided evidence of a relationship between the genetic variations rs10735781 and rs6680578 and MS in individuals of African-American ancestry [45]. This study concluded that the genetic variant rs10735781 influences modifications in the binding affinity of the PAX6 transcription factor. The transcription factor PAX6 is highly conserved and is crucial in neurogenesis and brain plasticity mechanisms [46]. The review highlights the significance of polygenic variations in MS, emphasizing their role in increasing the disease risk. It underscores the importance of understanding genetic and environmental factors due to the unknown cause of MS, aiming to uncover the disease's complex etiology. Recent research focuses on understanding genetic alterations linked to MS risk through studies, like GWAS, particularly the HLA system. Additionally, myelin-related genes and viruses, such as EBV, HHV-6, and measles, are discussed for their multifaceted roles in MS. The paper introduces PRS as a tool for assessing MS susceptibility, with a view toward personalized treatment. By confirming genetic findings in clinical practice and considering environmental factors, this work aims to enhance outcomes for MS patients, driving the patient-centered motivation behind these discoveries. Understanding the specific genes involved, their interactions, and the role of environmental factors is essential for elucidating the underlying causes of MS and potentially developing future personalized treatments.

## 2 GENETIC VARIANTS AND IMMUNE SYSTEM DYSREGULATION IN MS

### 2.1 Role of HLA region and HLA-DRB1*15:01 Allele

Although the HLA gene exerts a substantial hereditary influence on susceptibility to MS, the precise mechanisms by which it affects an individual’s likelihood of developing the disease remain uncertain. Researchers have investigated the relationship between MS and other autoimmune disorders using GWAS and serological typing techniques [27]. These methods have enabled the identification of numerous HLA-DR and DQ genes associated with MS development. However, Linkage Disequilibrium (LD) within the HLA region poses challenges in pinpointing the specific HLA gene linked to illness risk [47].

The HLA region, the Major Histocompatibility Complex (MHC), is located on chromosome 6p21 and spans a genomic area of 7.6 megabases. The scrutinized genomic region comprises 252 loci currently undergoing transcription, with a significant proportion associated with genes involved in immune response mechanisms [48]. The immune system’s importance is underscored by its ability to encode proteins critical for recognizing and presenting antigens to immune cells. Extensive research in the field of HLA in relation to MS has identified a particular allele, HLA-DRB1*15:01, showing a significant correlation with increased vulnerability to MS pathology [27]. The HLA region contains a cluster of genes responsible for encoding HLA proteins, which are categorized into two distinct classes: HLA class I (HLA-A, B, and C) and HLA class II (HLA-DR, DP, and DQ) [48].

HLA class II molecules are glycoproteins that form heterodimers on the cellular membrane. Their molecular structure comprises two distinct chains, α and β, encoded within the class II area of the MHC [49, 50]. These molecules are primarily synthesized by Antigen-presenting Cells (APCs), crucial in presenting antigens to CD4^+^ T cells, a specific subset of immune cells involved in modulating immune responses [51]. The presence of the class II association in the Northern European population, particularly in individuals with the DRB5*0101, DRB1*1501, DQA1*0102, and DQB1*0602 haplotypes, is well-documented. This strong association between HLA haplotypes is likely attributable to the functional epistatic interaction between the DRB1*1501 and DRB5*0101 alleles, as demonstrated by previous studies [52, 53]. The TNF (Tumor Necrosis Factor), complement components C3, C4, C5, and heat shock proteins have also been identified within the class III region [48].

GWASs have linked 233 frequently occurring genetic variations to MS; 32 are located inside the HLA area and 201 outside of it [54]. The HLA-DRB1 gene is significant in MS, recognized as one of the most pivotal genes within the HLA class II gene family. The HLA-DRB1*15:01 allele, located within the HLA-DRB1 gene, has consistently shown a notable hereditary susceptibility to MS onset. Enz *et al.* noted that the prevalence of this allele is higher among individuals diagnosed with the condition compared to the general population. Individuals with the HLA-DRB1*15:01 allele exhibit a three to four times greater vulnerability to MS onset than those without this genotype [55]. Approximately 60% of individuals diagnosed with MS inherit this allele, highlighting its significance in the disease context [56].

The HLA-DRB1*15:01 allele is hypothesized to influence MS development significantly through its modulation of immune system functioning. The exact mechanisms of this allele's effects are not fully understood; however, a hypothesis suggests that it might alter the presentation of specific antigens to immune cells. Consequently, an atypical immune response against self-antigens could occur, leading to inflammation and subsequent Central Nervous System (CNS) damage [48]. Research indicates that individuals carrying the HLA-DRB1*15 allele are more likely to develop MS at a younger age than those without this gene. Understanding the HLA region, particularly the HLA-DRB1*15:01 allele, is crucial for comprehending the immunogenetic basis of MS [57-59].

**Fig. (1):**
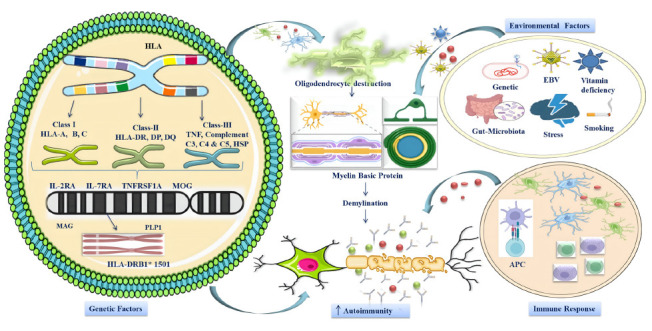
The interplay of genetic, immune, and environmental factors in MS pathogenesis. This diagram depicts the complex interaction among genetic, immune response, and environmental factors in the progression of MS. The diagram demonstrates that the aetiology of MS is complex. The figure illustrates how genetic predisposition, particularly in the MHC region, can impact MS risk. The genetic variants of HLA genes are divided into classes I, II, and III. A potent allele, HLA-DRB1*1501, is strongly associated with MS progression in class-II HLA genetic variants. The diverse environmental factors include EBV, vitamin D deficiency, smoking, gastric microbiome, stress, and location. These variables alter the function and activation of the immune system, initiating or aggravating MS symptoms. T cells, B cells, macrophages, and cytokines interact in a circular circuit during the immune response. The diagram illustrates how genetic predisposition and environmental factors can result in an abnormal immune response that infiltrates auto-reactive immune cells into the CNS. These cells demyelinate the myelin sheath, which protects nerve fibres, resulting in central inflammation and additional neurological impairments. In the development of MS, the figure highlights the complex interactions among genetic predisposition, environmental stimuli, and immune response dysregulation. It demonstrates that these three factors may contribute to oligodendrocyte degradation, MBP damage, and myelin damage, all of which increase the likelihood of developing the disease. **Abbreviations:** MS: Multiple sclerosis, MHC: Major histocompatibility complex, HLA: Human leukocyte antigen, EBV: Epstein Barr virus, HLA-DRB1*1501: Human leukocyte antigen class II, DR beta 1 precursor, MBP: Myelin basic protein.

Waubant *et al.* stated that an individual's MS susceptibility is influenced by various genetic and environmental factors and their interactions [60]. A well-functioning gut microbiota producing anti-inflammatory compounds can mitigate inflammation. In contrast, a gut microbiota imbalance, or dysbiosis, is associated with conditions, like endotoxemia, intestinal or systemic inflammation, and neuroinflammation [61-63]. Numerous studies have shown a positive correlation between stressful life events and a significant increase in MS aggravation likelihood in diagnosed individuals, typically manifesting within weeks or months of the stressful event [64-66]. Further research is required to elucidate the precise mechanisms by which these genetic factors contribute to MS initiation and progression. This understanding can enhance the precision of diagnosing, treating, and managing this neurodegenerative condition (Fig. **1**).

### 2.2 Genes Involved in Immune Response, Inflammation, and Myelin regulation

#### 2.2.1 Immune Response and Inflammation Regulating Genes

##### 2.2.1.1 IL-2RA (Interleukin-2 Receptor Alpha)

Gene involvement plays a vital role in various biological processes, including immune response, inflammation, and myelin remodeling [67]. Researchers have discovered that susceptibility to MS is linked to genetic variations in the IL-2RA gene, which influence the control and activation of T cells [68]. This gene is crucial in T cell activation; upon antigen exposure, T cells upregulate IL-2RA gene expression, enhancing IL-2RA's presence on the cell membrane. The high-affinity IL-2 receptor is formed by associating additional subunits, enabling T cells to detect and respond to IL-2 signals effectively [69]. The IL-2 receptor complex is pivotal in facilitating IL-2-mediated signaling, essential for T cell proliferation, survival, and differentiation. Additionally, IL-2RA production regulates immunological responses by balancing various T cell subsets, including regulatory T cells (Tregs) and effector T cells [69].

Buhelt *et al.* found that the Single Nucleotide Polymorphisms (SNPs) rs2104286 and rs11256593 in the IL-2RA gene correlate with CD25 expression in CD4^+^ T cells [70]. However, these SNPs show no significant association with CD25 expression in CD8^+^ T cells. The rs2104286 variation in the IL-2RA gene is identified as a genetic susceptibility factor for MS. A correlation between alterations in IL-2RA expression and T-cell dysregulation was established [71]. In a research study by Linder *et al.,* another IL-2RA gene variant, rs12722495, was linked to increased MS susceptibility. This study also assessed the variant's impact on IL-2RA protein expression and T-cell activity [72]. The genetic variations identified in studies by Peerlings *et al.* and Kumar *et al.* were found to play a significant role in predisposing individuals to MS by disrupting IL-2RA function and altering immune responses [68, 73]. However, it is crucial to remember that MS development is significantly influenced not only by genetic, but also by environmental factors [33]. The findings suggest a correlation between IL-2RA genetic variations and MS progression.

##### 2.2.1.2 IL-7R T2441 Polymorphism and MS

The Interleukin 7 Receptor (IL7R), classified as a type-I cytokine receptor, is characterized by alpha and gamma chains. This receptor is present in a significant proportion of activated adult T cells, thymocytes, and immature B cells up to the pre-B stage [74]. A study by Galarza-Munoz *et al.* found a correlation between certain variations of the IL-7R gene and increased susceptibility to MS [75]. The IL-7R gene encodes the interleukin-7 receptor protein, playing a crucial role in the development and viability of T cells, a leukocyte subset involved in immune responses [76]. Multiple studies have reported a correlation between genetic variations in the IL-7R gene, particularly the Single Nucleotide Polymorphism (SNP) rs6897932, and increased susceptibility to MS [11, 26, 77-79]. Individuals with genetic variations in the IL-7R gene, related to MS, exhibit dysregulation in T cell production and activity, leading to abnormal immune responses. This malfunction contributes to the inflammatory processes reported in MS [80].

SNP rs6897932, a genetic variant of IL-7R, has been extensively investigated. Its association with increased MS susceptibility has been established across several populations [81]. This variant is located on chromosome 5p13.8–15, specifically within exon 6 of the IL-7R gene, in a transmembrane region of the expressed protein. Linkage studies have shown a significant association between the 5p12–14 area of the chromosome and increased neurodegenerative disease susceptibility [82]. Exon six is frequently skipped due to non-conservative amino acid changes at position 244 (ILe-Thr) in the IL7R T244I variant [83]. The IL7R gene comprises eight exons, and alternative splicing produces a soluble isoform lacking exon six. Removing exon 6 alters the proportion between membrane-bound and soluble isoforms, increasing the percentage of messenger RNAs encoding the secreted receptor version, sIL7R. This change leads to elevated plasma sIL7R levels [84].

The rs6897932 genetic variant is an SNP, indicating a single base pair substitution in the DNA sequence. This modification could affect the IL-7R protein's structure or functionality, influencing T-cell proliferation and viability and potentially increasing disease susceptibility [28]. The IL-7R gene exhibits several new SNPs, including rs3194051, rs987107, and rs11567686 [79]. It is critical to understand that IL-7R genetic variations are just one of many factors that may contribute to MS onset and progression [85].

##### 2.2.1.3 TNFRSF1A (Tumor Necrosis Factor Receptor Superfamily Member 1A)

The gene TNFRSF1A encodes the protein known as TNF Receptor type 1 (TNFR1). This cell surface receptor binds to TNF, a cytokine implicated in the pathogenesis of numerous inflammatory disorders [86]. The TNFRSF1A gene is associated with various illnesses, including TNF Receptor-associated Periodic Syndrome (TRAPS), a rare autosomal dominant disorder [87]. Missense mutations in the TNFRSF1A gene are frequently implicated in the etiology of TRAPS. These mutations result in an aberrant TNFR1 protein, causing dysregulated signaling of the TNF-α cytokine and subsequent chronic inflammation [88]. Two common variations, R92Q (rs4149584) and P46L (rs1800693), are associated with TNFRSF1A and TRAPS. The R92Q variant involves substituting arginine with glutamine at position 92 within the TNFR1 protein and is frequently observed in individuals diagnosed with TRAPS [89-91]. The P46L mutation results in a substitution of proline with leucine at position 46 within the TNFR1 protein. This TRAPS variation, described by Zhao *et al.,* is widely observed [86].

In addition to TNFRSF1A variations in TRAPS, scholarly studies have explored the association between these variants and various inflammatory illnesses, including MS [90], rheumatoid arthritis [88], and inflammatory bowel disease [91]. TNFRSF1A variations have primarily been associated with the pathogenesis of TRAPS, characterized by recurrent episodes of fever and inflammation. However, a recent research work by Zegarska *et al.* suggests that these variants may also modulate the inflammatory response in MS [91]. Multiple studies have indicated that TNFRSF1A genetic variations can influence susceptibility to, or the progression of, MS [11, 87, 92]. TNF-TNFR1 interaction is essential for MS development and plays a significant role in inflammation, demyelination, cell death, and blood-brain barrier disruption [87, 92]. Two Single Nucleotide Polymorphisms (SNPs) in the TNFRSF1A gene are associated with MS. The SNP rs1800693 T/C, located in intron-6, is a common non-coding variant affecting the s-content>bound TNFR1 ratio, thus increasing sTNFR1 levels. The other SNP, rs4149584 G/A, in exon 4, is a rare coding polymorphism with a more significant effect on MS and its associated traits. This mutation impacts the contact region between TNF-α and its receptor, enhancing electrostatic contacts and interactions with the ligand, potentially leading to modifications in the receptor's signaling pathway [89, 93].

Based on the above data, it can be inferred that there is a lack of comprehensive understanding regarding the specific processes through which genetic variations contribute to MS. The interplay among genetic determinants, environmental triggers, and immune dysregulation in MS is complex and multidimensional. Further investigation is warranted to ascertain the specific involvement of TNFRSF1A mutations in the susceptibility and progression of MS.

#### 2.2.2 Myelin Regulation

##### 2.2.2.1 The MOG (Myelin Oligodendrocyte Glycoprotein) Gene

MOG, an Immunoglobulin (Ig) specific to the CNS, is localized exclusively on the outer surface of myelin sheaths and oligodendrocyte membranes. Eliseeva *et al.* and Peschl *et al.* reported that MOG, a significant biomarker associated with oligodendrocyte formation, may serve as a potential target for cellular and humoral immune responses in inflammatory demyelinating diseases, such as MS [94, 95]. Although the precise cause of MS remains uncertain, it is widely acknowledged that genetic factors significantly influence an individual's susceptibility to the disease. Investigations of variants or genetic polymorphisms in the MOG gene aim to ascertain their potential association with MS susceptibility [96]. These genetic variations affect the development and function of the MOG protein, potentially influencing immunological response and myelin tolerance [95].

Multiple studies have established a correlation between specific MOG gene variations and increased susceptibility to MS [97]. The polymorphisms examined include G15A, Val142Leu, 571 + 68 (IVS 4), and 571 + 77 (IVS 4) [98, 99]. A study observed that alleles associated with novel polymorphism, specifically Val 142 and 571 + 68A, were less frequent among individuals diagnosed with MS than those without the condition [98]. This finding suggests a potential association between these alleles and MS development. The prevalence of these variations may vary across different ethnicities [60]. Notably, while the immune system targets MOG in MS, it is not the only myelin protein implicated in the disease's pathogenesis. Other proteins involved in myelin formation, such as MBP and PLP, have also been observed as targets in MS [100]. The immunological response to these proteins elicits inflammation, demyelination, and brain function impairment [101]. Based on these findings, it may be concluded that the MOG gene is significantly associated with MS progression, a neurodegenerative illness. Further research is necessary to elucidate the role of MOG gene variations in MS susceptibility and progression.

##### 2.2.2.2 MAG (Myelin-associated Glycoprotein) Gene

Dysregulated MAG expression has been documented in MS lesions. MAG is crucial for preserving and reinforcing myelin, the protective sheath for nerve fibers in the CNS [102]. Predominantly expressed on the cellular membrane of oligodendrocytes, responsible for myelin formation, MAG's dysregulation in MS leads to CNS lesions and interferes with neural impulse propagation [103, 104].

A decrease in MAG levels is observed in MS lesions, particularly in demyelinated areas. This downregulation may adversely affect myelin maintenance and repair mechanisms [102]. MAG stabilizes myelin structure and facilitates connections between myelin and axons, the elongated projections of nerve cells. The defective remyelination process in MS is further influenced by altered MAG expression in lesions [105]. Remyelination, the regeneration of the myelin coating around damaged axons, is crucial for restoring normal nerve function. Alizadeh *et al.* noted that reduced MAG levels could impede the remyelination process and affect the restoration of compromised nerve fibers [106]. Understanding MAG's role and altered expression in MS lesions is vital for developing treatments that promote remyelination and improve impaired myelin restoration.

##### 2.2.2.3 PLP1 (Proteolipid Protein 1) Gene

PLP1 is a critical structural protein component in myelin, comprising approximately 50% of the protein content in CNS myelin. Notably, the amino acid sequence of PLP1 is perfectly conserved across human, mouse, and rat species, underscoring its vital role in myelin function. This observation is significant, especially considering that complete gene deletions have been linked to the least severe disease manifestations [107]. Cloake *et al.* reported a correlation between PLP1 gene variations and increased susceptibility to certain forms of MS. Mutations or alterations in the PLP1 gene may lead to abnormal PLP1 protein production and function [108]. These issues could potentially result in Pelizaeus-Merzbacher Disease (PMD), a rare neurological disorder characterized by deficient or impaired myelin in the CNS [109].

Interestingly, there is an overlap in the genetic risk factors for PMD and specific MS forms. While PMD and MS are distinct, studies have shown that mutations in the PLP1 gene can influence susceptibility to both conditions [110]. PLP1 gene variations have a low prevalence in the general population and are more strongly associated with familial or hereditary forms of MS [108]. The PLP1 locus, situated in the Xq22 region of the chromosome, spans 17 kilobases and comprises seven exons encoding the PLP1 protein. Exon 3 contains an internal splice site, producing a truncated alternative transcript for the DM20 isoform. Mutations affecting the amino acid sequences in PLP1 and DM20 are often considered harmful due to their high conservation across species [110].

The exact mechanism by which these mutations lead to clinical conditions remains unclear. However, in PMD animal models, PLP1 gene mutations cause oligodendrocyte death [108, 111, 112]. Likewise, cellular organisms transfected with PLP1 sequences containing established PMD mutations exhibit apoptosis due to PLP accumulation in the Endoplasmic Reticulum (ER) and subsequent activation of the Unfolded Protein Response (UPR) [108, 113, 114]. The UPR, a crucial biological process, prevents the accumulation of misfolded proteins. Translating CCAAT/enhancer binding protein Homologous Protein (CHOP) from the cytoplasm to the nucleus is a known marker of UPR activation [115, 116]. These significant findings suggest a strong link between PLP1 gene variations and the development of neurodegenerative conditions, like MS. Further research is needed to clarify how PLP1 gene variations contribute to MS pathogenesis in order to develop targeted therapeutic strategies.

## 3 NON-HLA GENETIC VARIANTS AND MS RISK

### 3.1 Overview of Non-HLA Susceptibility Loci Identified Through GWAS

GWASs have been instrumental in identifying non-HLA genetic variations associated with MS risk. The HLA genes, crucial for immune system functionality, have a well-recognized link to MS susceptibility [117]. Moreover, it is noteworthy that non-HLA genes also significantly contribute to MS development. Utilizing GWAS, researchers have identified several non-HLA susceptibility loci for MS. GWAS involves a comprehensive analysis of genetic variations in individuals with a specific disease and in unaffected individuals, aiming to identify genetic differences more prevalent in the disease-affected group [11].

#### 3.1.1 CD40 (Cluster of Differentiation 40)

The CD40 gene, called cluster of differentiation 40, is crucial in immunological signaling, particularly in activating B cells responsible for antibody production and immune regulation [118]. Field *et al.* discovered genetic variations in the CD40 gene to be linked to increased MS susceptibility [119, 120]. Aarts *et al.* suggested that these variations might affect CD40 protein expression or function, altering immune responses and possibly influencing neurodegenerative disease progression [121].

CD40 is primarily expressed on B cell membranes, interacting with its ligand, CD40L, on activated T cells. This interaction is essential for initiating B cell activation and antibody synthesis. The CD40-CD40L signaling pathway modulates immune responses, activating other immune cells and releasing pro-inflammatory mediators [118]. Disrupted CD40-mediated immune responses may contribute to the autoimmune mechanisms observed in MS [120]. Abnormal B and T cell interactions and atypical antibody production are linked to MS pathophysiology [122]. The importance of CD40 variations lies in their association with several autoimmune disorders, such as Systemic Lupus Erythematosus (SLE) and Rheumatoid Arthritis (RA), suggesting shared genetic and immunological dysregulation mechanisms [123]. Elgueta *et al.* found a correlation between the MS risk allele at rs1883832 and increased CD40 expression, potentially leading to a heightened pro-inflammatory response [118].

In conclusion, there is a significant link between genetic diversity in CD40 and MS progression. Further research is necessary to understand how CD40 genetic variations influence MS susceptibility, B cell function, and immune responses during the disease. Insights into CD40's role in MS etiology could lead to novel therapeutic strategies targeting immune response modulation and disease progression inhibition.

#### 3.1.2 CD58 (Cluster of Differentiation 58)

CD58, often called cluster of differentiation 58 or Lymphocyte Function-associated Antigen 3 (LFA-3), is a critical genetic element in the immune response, enhancing intercellular connections among immune cells. The 2021 study by Zhang *et al.* identified a correlation between genetic variations in CD58 and increased susceptibility to MS. CD58 expression is primarily observed on the cell membranes of Antigen-presenting Cells (APCs), such as macrophages and dendritic cells [124]. It is also present in T and Natural Killer (NK) cells. CD58 interacts molecularly with its corresponding receptor, CD2, in T and NK cells, facilitating cellular adhesion and signal transmission across immune cells [124-126]. The CD58 gene locus is a significant risk factor for MS beyond the HLA region, as indicated by studies [127, 128]. Single Nucleotide Polymorphisms (SNPs) in the first intron of the CD58 gene, specifically rs12044852, rs10801908, rs1335532, and rs2300747 (as identified in the dbSNP database), show a strong association with MS. The association's strength, measured by odds ratios, varies across studies. The multinational GWAS by Beecham *et al.* and Patsopoulos *et al.* reported an odds ratio of 1.30 [129, 130]. In contrast, a regional cohort study in Germany by De Jager *et al.* found an odds ratio of 2.13 [125]. In familial MS cases, compared to controls, D'netto *et al.* reported an odds ratio of 2.63 [131].

Hecker *et al.'s* 2019 study revealed genetic variations within the CD58 gene correlated with increased susceptibility to MS based on GWAS [132]. These variations significantly affect CD58 protein expression and functionality, presumably leading to changes in immunological responses implicated in MS development [124]. The CD58-CD2 connection is vital in activating and regulating T-cell responses, including T-cell adhesion, IL-12 signaling, co-stimulation, and cytokine production. Zhang *et al.* noted that CD58 malfunctions could lead to reduced intercellular communication among immune cells, poor T-cell activation, and immunological response dysregulation [124]. Genetic variations in CD58 might disrupt immune cell adhesion and communication, compromising the ability to regulate immunological responses and increasing MS susceptibility [133].

**Fig. (2):**
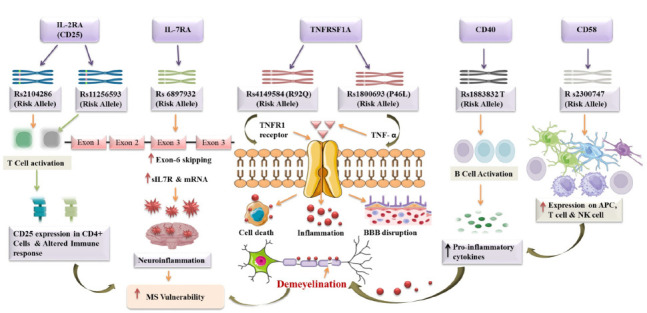
Role of genetic variants in the progression of MS. The graphic diagram illustrates the significant role of specific genetic variations in advancing MS. The haplotypes rs2104286, rs11256593, rs6897932, rs4149584, rs1800693, rs1883832 T, and rs2300747 are associated with important immune-related genes. The discovered SNPs have been found to play a role in controlling the immune response, leading to alterations in the severity and progression of MS. The immunological regulation and cell communication processes require the participation of several molecules, including IL-2RA, IL-7RA, TNFRSF1A, CD40, and CD58. The capacity of genetic sequences to influence the functioning of immune cells, including T cells and antigen-presenting cells, can exert an effect on the pathogenesis and advancement of MS. Specific alleles within these genes, namely rs2104286 and rs11256593, induce T cell activation and CD25 expression in CD4+ cells, along with a modified immunological response, resulting in heightened inflammation and subsequent demyelination. The IL-7RA allele rs6897932 is associated with exon-6 skipping, leading to increased levels of IL-7R in the bloodstream and mRNA. This molecular alteration has been implicated in the development of neuroinflammation and demyelination. The interaction between Tumour Necrosis Factor (TNF) and its receptor TNFR1 leads to the activation of inflammatory responses, cell death, and disruption of the Blood-brain Barrier (BBB). These processes have a role in the evolution of MS. The genetic variations rs4149584 and rs1800693 in the TNFRSF1A gene are responsible for encoding the TNFR1 protein. The genetic variant rs1883832 represents a polymorphism located within the CD40 gene. T activation elicits a response in immune cells, leading to an increase in the production of pro-inflammatory cytokines. The genetic variant rs2300747 has been identified as a CD58 variant that has been found to enhance the presence of several immune cells, such as APC cells (including macrophages and dendritic cells), T cells, and NK cells. This variant is associated with an overexpression of pro-inflammatory cytokines, disrupting the immune system. Consequently, this disruption contributes to inflammation, demyelination, and neuronal damage characteristic of MS. **Abbreviations:** IL-2RA: Interleukin-2 receptor alpha, CD40: Cluster of differentiation 40, TNF: Tumor necrosis factor, TNFR1: Tumor necrosis factor receptor 1, APC: Antigen-presenting cells, BBB: Blood-brain barrier, NK: Natural killer cells.

Previous research has linked CD58 variations to other autoimmune disorders, including Rheumatoid Arthritis (RA) and Systemic Lupus Erythematosus (SLE), suggesting common genetic determinants and immunological dysregulation across these diseases [134]. A study observed an association between the rs2300747G allele and increased CD58 mRNA expression in lymphoblastic cell lines and peripheral blood mononuclear cells from MS patients. This increase was allele dosage-dependent. De Jager *et al.* found elevated CD58 mRNA expression levels in MS patients during clinical remission, suggesting a beneficial effect of increased CD58 expression on circulating mononuclear cells [125]. These findings indicate a potential link between CD58 gene variants and MS. Further research is necessary to fully understand how CD58 genetic variations affect MS susceptibility and their impact on immune cell interactions and responses in MS. Understanding CD58's role in MS pathogenesis may illuminate potential therapeutic targets for modulating immune cell interactions and regulating the immune system in MS (Fig. **2**).

#### 3.1.3 CLEC16A (C-type Lectin Domain Family 16, Member A)

The gene CLEC16A, also known as 'C-type lectin domain family 16 member A', plays a significant role in the modulation and functioning of immune cells [135]. Genetic variations in the CLEC16A gene have been linked to the development of autoimmune disorders, such as MS. However, a comprehensive understanding of its functional significance remains elusive [136]. The CLEC16A gene exhibits expression in various immune cell types, including B, T, and dendritic cells. Subsequent studies underscore the pivotal role of this gene in immune cell signaling, antigen presentation, and the regulation of immune responses [135, 137].

A research study conducted in Norway demonstrated a significant association between the CLEC16A Single Nucleotide Polymorphism (SNP) rs12708716 and MS. Further investigation revealed a robust correlation between the rs12708716 genotype and the relative expression of two distinct CLEC16A transcripts in the thymus, but not in blood. This finding suggests the presence of thymus-specific or cell-specific splicing regulation [138, 139]. GWASs have identified genetic variations in the CLEC16A gene as potential risk factors for the development of MS. According to Fan *et al.,* these variations may affect the expression or function of CLEC16A, potentially leading to immune response dysregulation and increased susceptibility to MS [140]. Additionally, current research indicates that CLEC16A may regulate autophagy, a vital cellular process crucial for maintaining cellular balance and eliminating damaged or dysfunctional components [137, 140].

The dysregulation of autophagy has been implicated in certain autoimmune diseases, including MS [141]. Moreover, associations have been observed between CLEC16A polymorphisms and alterations in immune cell functionality, such as impaired signaling pathways and reduced antibody production. The role of B cells in MS pathogenesis is well-documented; they contribute to disease progression through autoantibody production and modulation of immune responses [80, 134]. In light of these findings, there appears to be a strong correlation between CLEC16A variations and the progression of MS. Furthermore, a deeper understanding of CLEC16A's role in MS susceptibility could provide valuable insights into the underlying immune dysregulation and inform potential therapeutic targets for treatments aimed at modulating immune responses and mitigating disease progression.

#### 3.1.4 IRF8 (Interferon Regulatory Factor 8)

A study conducted by Salem *et al.* demonstrated that IRF8 plays a crucial role in regulating immune cell activation, differentiation, and the generation of pro-inflammatory cytokines. The gene IRF8 contributes to the control of the immune response [142, 143]. The presence and activity of immune cells, particularly those of the myeloid lineage, such as monocytes, macrophages, and dendritic cells, are crucial for the proper development and operation of the immune system [144]. Genetic variations in the IRF8 gene have been identified as being associated with increased vulnerability to MS. A study revealed a correlation between the rs17445836G variant in individuals diagnosed with secondary progressive MS and decreased serum type I Interferon (IFN-1) concentration. Moreover, this genetic variation, rs17445836G, was notably associated with increased levels of IRF8 expression in B cells among individuals diagnosed with Systemic Lupus Erythematosus (SLE) [145].

The expression and function of IRF8 can be influenced by these genetic variations, potentially resulting in immunological dysregulation and increased susceptibility to neuroinflammatory disorders, such as MS [146]. IRF8 is essential in proliferating immune cells within the Central Nervous System (CNS) and demonstrates connections with peripheral cells [147]. Malfunction of IRF8 has been implicated in the dysregulation of immunological responses and the development of autoimmune disorders, including MS [143, 146, 148]. IRF8 is involved in the process of myeloid cell differentiation and the production of cytokines that regulate the equilibrium between pro- and anti-inflammatory responses [149].

Furthermore, previous studies have established a correlation between IRF8 and the activation and control of microglia, immune cells in the CNS crucial for neuroinflammation and immunological reactions within the brain and spinal cord [150]. The pathogenesis of MS is thought to involve the dysregulation of microglia activation and function [151]. The precise mechanisms by which genetic differences in IRF8 contribute to susceptibility to MS and the specific roles of IRF8 in modulating immune cells and the pathogenesis of MS have yet to be fully elucidated [27, 152]. Based on the findings of this significant research, it can be inferred that there exists a strong correlation between the genetic variant of IRF8 and the development of MS. Further research is necessary to comprehend the functional implications of IRF8 in the progression of MS and identify potential therapeutic interventions targeting this pathway.

### 3.2 Genes Associated with T-cell Activation, B-cell Function, and Immune Cell Migration

#### 3.2.1 CXCR5 (C-X-C Motif Chemokine Receptor 5)

The chemokine receptor CXCR5, expressed on B cells and T follicular helper (Tfh) cells, facilitates migration to lymphoid organs. This migration is crucial for the interaction between B cells and Tfh cells, which leads to antibody production and the initiation of immunological responses. Previous studies have established a link between CXCR5 variants and susceptibility to MS [153, 154]. Gil-Varea *et al.* suggested that genetic variations may affect the expression or functionality of CXCR5, potentially disrupting immune cell migration and altering immunological responses associated with MS. The use of peripheral blood mononuclear cells from MS patients for immunophenotyping revealed a significant association between the minor allele of rs10892307 and increased Tregs expressing CXCR5, suggesting a potential connection between this polymorphism and MS, possibly due to the increased presence of circulating CXCR5-expressing Tregs [155].

In MS, CXCR5 plays a vital role in developing and maintaining immune cell aggregates within lymphoid tissues, such as lymph nodes and the spleen. These aggregates, known as germinal centers, are sites where B cells undergo activation, proliferation, and differentiation into plasma cells that produce antibodies [156, 157]. Tfh cells are essential in supporting B cells in germinal centers, facilitating antibody synthesis and the development of a precise, focused immune response [158]. CXCR5 is critical in directing Tfh cell movement towards B-cell zones within lymphoid organs, enabling effective engagement and cooperation with B cells in their microenvironments. Dysregulation in CXCR5 expression or activity may interfere with the proper migration and localization of B and Tfh cells within lymphoid organs, impairing their ability to interact and coordinate immune responses. Such dysregulation could affect MS development or progression by influencing antibody production and immunological control [158, 159]. Further research is necessary to elucidate how genetic variations in CXCR5 contribute to MS susceptibility and their impact on immune cell migration and immune responses within the context of the disease. A deeper understanding of CXCR5's role in MS pathogenesis could illuminate potential therapeutic strategies to modulate immune cell migration and optimize immune responses in MS.

#### 3.2.2 CCR5 (C-C Chemokine Receptor Type 5)

The CCR5 receptor, a chemokine receptor, facilitates the migration of immune cells, particularly T lymphocytes, to sites of inflammation [160]. Known as C-C chemokine receptor type 5, CCR5 is prominently located on the cellular membrane of immune cells, including T cells and macrophages. This receptor significantly influences the migration of immune cells to inflammatory areas in the CNS [160, 161]. Genetic variations in the CCR5 gene are associated with susceptibility to MS, and changes in CCR5 expression impact immune cells' movement and the MS's inflammatory response [162, 163]. The study by Ellwanger *et al.* discussed genetic variations that influence CCR5 expression, potentially affecting immune cell trafficking and the inflammatory response in MS [164].

Furthermore, the CCR5 gene plays a role in modulating chemokine-mediated immune cell motility. It interacts with chemokines, such as CCL3, CCL4, and CCL5, implicated in inflammation and immune responses [165, 166]. Alterations in CCR5 expression or functionality can induce changes in T cell migration to the CNS, influencing their infiltration and contributing to the inflammatory response in MS lesions [167].

The genetic variations, including the CCR5-Δ32 deletion variant, have been linked to the modified expression and functionality of CCR5. According to Ellwanger *et al.,* the CCR5-Δ32 variant results in a truncated, non-functional receptor, reducing T-cell migration to inflammation sites. The CCR5 gene's diverse functions in MS and the impact of genetic variations on disease susceptibility and progression vary among ethnic groups [164]. Previous research indicated that individuals with a mutant allele were resistant to HIV-1 infection or experienced slower AIDS progression, demonstrating a protective effect. However, a CCR5 mutation in MS patients was associated with earlier mortality [168, 169].

Additional research is essential for a comprehensive understanding of the impact of CCR5 genetic variations on MS susceptibility and their influence on immune cell trafficking and inflammation. Understanding the role of the CCR5 gene and examining the dynamics of cytokine-cytokine and cytokine-neurotransmitter interactions is crucial. The CCR5 mutation shows potential as a prognostic indicator and could inform the development of therapeutic strategies for individuals with MS.

#### 3.2.3 CTLA4 (Cytotoxic T-Lymphocyte-Associated Protein 4)

CTLA4 (Cytotoxic T-lymphocyte-associated protein 4) is a co-inhibitory receptor on activated T cells. It regulates T-cell activation and immune responses, with abnormalities in CTLA4 signaling potentially leading to immune dysregulation in MS [170]. Competing with the co-stimulatory receptor CD28 for binding sites on antigen-presenting cells' B7 molecules (CD80 and CD86), CTLA4 transmits inhibitory signals to T cells, reducing activation and attenuating immune responses [171].

The association between CTLA4 gene variations and increased MS susceptibility has been established. CTLA4 signaling or expression failures may contribute to MS's immunological dysregulation [172]. Although the exact mechanism by which CTLA4 variations affect MS is unclear, it is hypothesized that abnormal CTLA4 signaling disrupts the balance between effector T cells and Tregs in MS, leading to intensified immune responses and increased inflammation [173]. Notable mutations in the CTLA-4 gene include those in the 3′ Untranslated Region (UTR), one in the promoter region, and one in the first exon. Common allele combinations are 319C/T (rs5742909), +49A/G (rs231775), (AT)n, CT60A/G (rs3087243), and Jo31G/T (rs11571302). Experimental studies suggest that each polymorphic location correlates with susceptibility to autoimmune diseases or changes in immune responses. The T allele at 319C/T in the promoter region is associated with increased promoter activity compared to the C allele [174, 175].

**Fig. (3):**
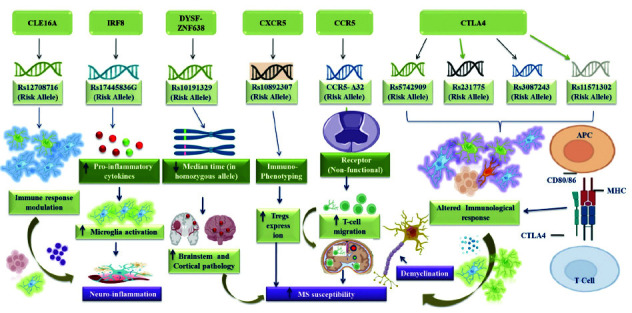
Genetic variants associated with T-cell activation, B-cell function, and immune cell migration in MS progression. The diagram depicts the notable contributions of distinct genetic variations to the development of MS. The genetic variations included in this set comprise the haplotypes rs12708716, rs17445836G, rs10191329, rs10892307, CCR5-32, rs5742909, rs231775, rs3087243, and rs11571302. Every variant is correlated with specific elements of MS progression. The genetic variant CLE16A (rs12708716) has been associated with the control of the immune system. It can potentially affect the inflammatory response, which plays a role in the development and progression of MS. The genetic variant IRF8 (rs17445836G) regulates immune responses and can potentially influence the equilibrium between pro-inflammatory and anti-inflammatory signals in MS. The genetic variant DYSF-ZNF638 (rs10191329) can affect the mechanisms involved in brain repair, potentially altering the remyelination process and the integrity of neurons. These factors are known to have significant implications for the progression of MS. The genetic variant CXCR5 (rs10892307) has been found to correlate with the movement and positioning of B-cells inside the CNS. This relationship has the potential to impact the development of inflammatory lesions that are commonly associated with MS. The CCR5 (CCR5-Δ32) gene variant is linked to the migration of immune cells. It potentially impacts the infiltration of immune cells into the CNS, hence playing a role in the development of chronic inflammation observed in MS. The variations rs5742909, rs231775, rs3087243, and rs11571302 of the CTLA4 gene are associated with immunological modulation and T-cell responses. The modulation of CTLA-4 signalling can potentially affect the equilibrium between immunological activation and repression, exerting an influence on the overall progression of MS. **Abbreviations:** IRF8: Interferon-regulatory factor 8, DYSF-ZNF638: Dysferlin zinc finger protein 638, CXCR5: C-X-C chemokine receptor 5, CCR5: C-C chemokine receptor 5, CTLA4: Cytotoxic T-lymphocyte–associated antigen 4.

These examples illustrate genes associated with T-cell activation, B-cell activity, and immune cell migration in MS. Each gene plays a role in various immune responses. Their complex interactions influence MS development. Further investigation is needed to fully understand how these genes contribute to MS pathophysiology and explore their potential as therapeutic targets (Fig. **3**).

#### 3.2.4 TNFAIP3 Gene

Tumor Necrosis Factor Alpha-induced Protein 3 (TNFAIP3), commonly known as A20, plays a crucial role in the regulatory mechanisms of the NF-κB signaling pathway, acting as a key component in its negative feedback control [176]. This molecule is the second component of the TNF-α pathway and is associated with the pathogenesis of various autoimmune disorders [177]. Analysis has revealed a genetic variant, rs10499194, located in the intergenic region upstream of TNFAIP3. Furthermore, the ENCODE project's *in vitro* ChIP-seq dataset demonstrates its localization within specific binding sites for several transcription factors, including JunD, BAF155, and DNase hypersensitive sites in RRMS and SPMS [178]. These significant findings suggest a link among TNFAIP3, its variant rs10499194, and neurodegenerative disorders, such as MS.

#### 3.2.5 CD226 Gene

Regarding the CD226 gene, the presence of a nonsynonymous mutation, Gly307Ser (rs763361), has been correlated with various autoimmune diseases, including MS. This finding supports the arguments of Gross *et al.* [179, 180]. Hafler *et al.* observed that this mutation may contribute to an increased activation threshold of NK cells due to the reduced expression of CD226 [180]. Clinical studies have shown that genetic variations can influence gene expression and increase disease susceptibility. Gross *et al.* reported decreased CD226 expression in MS [181-183]. It was found that rs763361 significantly affects CD226 expression in various biological samples [181]. Regulatory T cells from mice lacking CD226 exhibited reduced inhibitory function, leading to accelerated progression of experimental autoimmune encephalomyelitis, a model for MS [182]. These studies indicate a vital role of CD226 in regulating T cell activation and suggest that dysregulation of CD226 impairs the function of regulatory T cells [183, 184]. Therefore, rs763361 is potentially associated with neurological disorders, including MS.

#### 3.2.6 TYK2 Gene

In the TYK2 gene, the association of an SNP, rs34536443, which results in the substitution of proline for alanine at amino acid 1104, has been established through GWAS. This mutation offers protection against various autoimmune diseases, including SLE and MS [184]. However, the exact mechanisms by which this SNP provides protective effects in autoimmune pathogenesis are not fully understood. Gorman *et al.* observed a reduction in Tfh cells, altered memory B cells, and diminished IFNAR signaling in individuals carrying the protective variant TYK2A1104 (TYK2P) who had no pre-existing health conditions [185]. Additionally, a meta-analysis by Tao *et al.* confirmed the association of TYK2 SNPs rs2304256 and rs34536443 with MS, RA, SLE, Crohn's Disease (CD), and Ulcerative Colitis (UC) [186].

TYK2, a member of the Janus Kinase (JAK) family of tyrosine kinases, has been identified as a potential candidate gene associated with autoimmune illnesses, particularly those with hereditary factors. This association is attributed to TYK2's role in mediating cytokine signaling pathways, including those of Interferon type I (IFN-I) [187, 188]. As a non-receptor protein, TYK2 exhibits binding affinity for the inactive conformation of the IFN-I Receptor (IFNAR1) located on the cellular membrane. Upon Interferon-alpha (IFN-α) binding to IFNAR1, Signal Transducers and Activators of Transcription (STAT) 1 and 2 undergo phosphorylation. This event triggers the activation of both TYK2 and JAK1 proteins. Consequently, the regulation of numerous IFN-stimulated genes is controlled by STAT1/2 heterodimers within the nucleus [187, 189].

Autoimmune illnesses frequently correlate with abnormal production of IFN-I, other cytokines, or members of the JAK kinase family by immune cells [187, 190, 191]. TYK2 significantly influences various immunological processes, including the activity of NK cells, the growth of B and Treg cells, and the differentiation of Th1 and Th17 cells. Moreover, TYK2 is pivotal in IFN-I and other type I and II cytokine receptor pathways. Dysregulated expression of TYK2, associated with autoimmune illnesses, has been evidenced in multiple studies [191, 192]. These noteworthy discoveries imply a significant correlation between genetic variations in the TYK2 gene and the pathways involved in MS progression. The importance of TYK2 in autoimmune mechanisms is further highlighted by substituting proline with alanine at amino acid position 1104. This mutation confers protection against autoimmune disorders by reducing Tfh cells, altering the phenotype of memory B cells and modulating IFNAR signaling.

#### 3.2.7 SLAMF1 (Signaling Lymphocytic Activation Molecule Family Member 1)

**Fig. (4):**
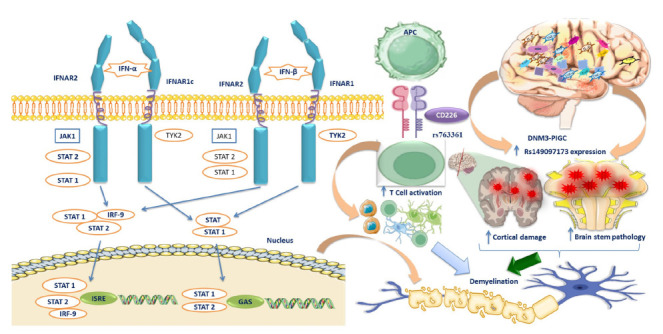
Role of numerous genetics variants and their pathways in the progression of MS. The diagram depicts the correlation among the TYK2 gene Single Nucleotide Polymorphism (SNP) rs34536443, the CD226 SNP rs763361, and the DNM3-PIGC SNP rs149097173 in the context of neurodegenerative diseases, such as MS. TYK2, belonging to the Janus Kinase (JAK) family, has been identified as a potential hereditary candidate gene associated with autoimmune disorders due to its role in regulating signalling pathways for various cytokines, particularly type I interferon. The non-receptor protein TYK2 interacts with the inactive IFN-I Receptor (IFNAR1) located on the cellular membrane. The process of IFN-binding to IFNAR1 leads to the phosphorylation of STAT 1 and 2, activating the TYK2 and JAK1 proteins. Many genes triggered by Interferons (IFNs) are subject to tight regulation by heterodimers of STAT1 and STAT2 within the nucleus. Autoimmune diseases often arise due to aberrant production of Interferon type I (IFN-I), other cytokines, or members of the JAK kinase family by immune cells. TYK2 influences various cellular processes, including but not limited to its involvement in the IFN-I and other type I and II cytokine receptor pathways. Additionally, TYK2 has a role in modulating natural killer cell activity and regulating the production of B and Treg cells. Furthermore, TYK2 is involved in the differentiation of Th1 and Th17 cells. There exists a correlation between dysregulated expression of TYK2 and autoimmune diseases. The CD226 genetic variant rs763361 holds significance in the activation of regulatory T cells, which are pivotal in the aberrant regulation that contributes significantly to the etiopathogenesis of MS. T lymphocytes undergo activation inside the lymphatic system and subsequently infiltrate the CNS utilizing arterial circulation in the context of MS. Upon arrival, T cells secrete pro-inflammatory cytokines, thereby inducing inflammation and resulting in tissue damage. The process described results in the degradation of myelin, nerve fibres, and the oligodendrocytes responsible for myelin production. A potential relationship was identified between rs149097173 in the DNM3-PIGC gene and significant genetic enrichment in CNS tissues. Elevated expression of the rs149097173 allele was found to be associated with cortical and brain-stem damage, subsequently leading to demyelination and an increased risk of MS. **Abbreviations:** TYK2: Tyrosine kinase 2, JAK: Janus kinase, DNM3-PIGC: Dynamin 3 phosphatidylinositol glycan anchor biosynthesis class C, IFN-1: Interferon-1, Th17: T-helper cells 17.

SLAMF1 (Signaling Lymphocytic Activation Molecule Family member 1) has also been studied for its genetic polymorphisms. Investigations into the SLAMF1 gene locus have revealed the minimal functional impact of the rs11265455 polymorphism's minor and major variants on the SLAMF1 promoter. However, the minor variant of the rs3753381 polymorphism in enhancer E has demonstrated a more than twofold increase in SLAMF1 promoter activity [192, 193]. This allelic variant significantly affects the binding of nuclear protein families, such as FOX, RXR, and NFAT. Putlyaeva *et al.* observed the expression of specific members of these families, including HNF4G, RXRB, and FOXO2 in MP-1 cell lines, as well as NFATC/3 and NR2C1 in Raji cell lines [192]. Yigit *et al.* reported that MS patients showed a higher proportion of T cells expressing SLAMF1 in their bloodstream [194]. However, the exact implications of this finding for the disease's progression and manifestation remain unclear. These results suggest a potential link between SLAMF1 genetic variations and increased susceptibility to MS (Table **1**, Fig. **4**) [195-197].

## 4 POLYGENIC RISK SCORES (PRS) AND PREDICTION MODELS IN MS

### 4.1 PRS and their Calculation

PRS are statistical methods that use an individual's genetic profile to forecast their hereditary susceptibility to a particular trait or ailment. The ability of genetic variants to predict disease susceptibility, aid in risk stratification, and facilitate personalized medicine has garnered significant attention in both genetic research and clinical applications [198]. The concept of PRS is grounded in the recognition that intricate traits and diseases, such as cardiovascular disease, diabetes, and psychiatric disorders, are impacted by the cumulative impacts of several genetic variants dispersed across the entirety of the genome. SNPs are prevalent genetic variations that manifest at specific loci within the DNA sequence [76]. Based on the aforementioned data, it can be concluded that PRS play a crucial role in predicting the strong correlation between genes and neurodegenerative diseases, specifically MS.

#### 4.1.1 Calculation of PRS

##### 4.1.1.1 Selection of GWAS Summary Statistics

PRS are commonly obtained through extensive GWAS, wherein many SNPs distributed throughout the genome are examined for their correlation with the specific characteristic or disease under investigation [25]. The summary statistics derived from GWAS provide valuable insights into the strength of the association between each SNP and the trait of interest. These statistics are subsequently utilized to calculate the PRS [199].

##### 4.1.1.2 Selection of the SNP Set and Effect Sizes

The selection of SNPs for inclusion in the PRS computation is based on a subset derived from the GWAS summary statistics. SNPs are commonly chosen following a predetermined threshold of significance, such as a p-value, or by considering patterns of LD [199]. The effect size estimates for each chosen SNP are acquired from the GWAS, as generally represented by beta or log-odds ratios [200].

##### 4.1.1.3 Weighting of SNPs

The impact sizes of the chosen SNPs are subsequently assigned weights based on their level of correlation with the specific trait or condition. The determination of weighting can be derived from several factors, such as the magnitude of the effect size, the significance level, or other pertinent metrics [201, 202]. The purpose of weighting is to provide further weight to SNPs that have a greater effect on the variable [203].

##### 4.1.1.4 Calculation of PRS

The calculation of the PRS involves the summation of weighted effect sizes for selected SNPs across all SNPs considered for an individual. The formula utilized for the computation of PRS is as follows:

PRS = Σ (weighted effect size * genotype) + intercept

In this context, “genotype” refers to an individual's count of risk alleles (0, 1, or 2) for each SNP. At the same time, the “intercept” denotes an optional constant value incorporated into the score [199].

### 4.2 Utility of PRS in Assessing Individual MS Risk

The utilization of PRS has been identified as a viable approach for assessing an individual's susceptibility to developing various diseases during their lifespan. This methodology has been particularly effective in evaluating the likelihood of obtaining MS based on an individual's genetic composition. The PRS is a method that considers the collective impact of many genetic variations associated with MS and provides a measure of an individual's genetic susceptibility to the disease [204].

#### 4.2.1 Predictive Power

The field of behavioral sciences has witnessed significant growth in the study of polygenic scores over the past decade [205]. PRS have demonstrated considerable predictive capacity in assessing an individual's likelihood of developing neurodegenerative conditions, such as MS. Hone *et al.* illustrated the use of a risk score to evaluate an individual's genetic susceptibility to diseases, like MS. This risk score, derived from multiple genetic variations linked to the disease's development, indicates a person's genetic predisposition to MS; a higher PRS suggests an increased genetic risk, while a lower PRS implies a reduced vulnerability [206]. Individuals with a genetic predisposition, as shown by the PRS, exhibit a significantly increased cumulative absolute probability of developing MS from age 20 onwards [206, 207]. Although PRS offers valuable predictive insights, it does not guarantee the occurrence of MS, a complex condition influenced by genetic and environmental factors. In contrast, PRS encapsulates only the genetic aspect of the disease [208]. For a comprehensive assessment of an individual's risk for MS, it is essential to combine PRS with clinical evaluation and other relevant risk factors [33].

The accuracy of polygenic score predictions depends on the Genotype-Environment (GE) relationship and population stratification. Over four decades, numerous quantitative genetic studies have examined the interplay between genetics and environmental factors. These studies have consistently revealed that a significant portion, approximately 25%, of the environmental measurements commonly used in behavioral sciences exhibit heritability [209-212]. Moreover, about 50% of these associations are influenced by genetic factors [213]. Earlier research has explored the dynamics of passive, evocative, and active gene-environment correlations [209]. Infants experience passive gene-environment correlations when they inherit environments that are genetically related. Parents with high polygenic Educational Attainment (EA) scores often pass these scores to their children, contributing to the heritability of EA. These parents also provide educational experiences that foster EA development, such as enrolling their children in tuition programs, setting ambitious goals, and providing positive role models. High EA scores may also prompt teachers to strive for improved academic outcomes. Active gene-environment correlation occurs when children select, modify, or create environments that align with their genetic predispositions. Children with advanced executive functioning skills often choose peers with similar interests, show greater classroom engagement, and engage more in reading activities. While passive GE correlation is confined to genetically related individuals, evocative and active correlations extend to interactions with non-related individuals [205]. Based on these findings, PRS is conclusively effective in predicting an individual's genetic susceptibility to diseases. Numerous studies have consistently shown that individuals with higher PRS are more prone to developing specified illnesses.

#### 4.2.2 Early Identification

The timely detection of individuals with an increased susceptibility to developing MS is paramount for adopting effective preventative strategies and enhancing condition management. Polygenic Risk Scores (PRS) have proven effective in the early detection of MS by evaluating an individual's genetic makeup and predicting their disease susceptibility, even before observable clinical symptoms manifest [214]. This assessment is valuable due to the substantial influence of genetic factors on MS susceptibility, with PRS capturing the cumulative impact of numerous genetic variants. The current technique can estimate an individual's susceptibility to MS before symptom manifestation. By examining genetic composition and calculating PRS, it is feasible to identify individuals with elevated MS susceptibility, even without clinical symptoms. Slunecka *et al.* noted that preclinical risk estimation facilitates timely detection and action [208].

Utilizing information about an individual's increased genetic susceptibility, healthcare professionals can implement preventative strategies and lifestyle adjustments, potentially delaying or mitigating MS occurrence [215]. High-risk individuals may be advised to increase their vitamin D levels, adopt healthy lifestyles, and avoid known environmental triggers. Alfredsson and Olsson suggested that high-risk individuals, as identified by PRS, can benefit from personalized monitoring approaches. Systematic assessments and ongoing surveillance of high-risk individuals can lead to early identification of MS signs and symptoms [33]. Ginsburg *et al.* stated that this can enable prompt clinical assessment and diagnostic investigation, allowing for timely intervention and therapy initiation, if necessary [216]. Filippi *et al.* argued that using PRS for early detection can expedite the administration of Disease-modifying Therapies (DMTs) when appropriate [217].

Research has shown that DMTs can slow MS progression and decrease relapse occurrence in affected individuals. Early treatment initiation, guided by heightened genetic susceptibility, can improve therapeutic outcomes and potentially reduce long-term impairment [218]. Cross *et al.* indicated that using PRS for early identification can enhance the selection of high-risk individuals for research and clinical trials [219]. This approach allows researchers to study MS's natural progression, evaluate new therapies, and test preventive measure efficacy within a high-risk group [220]. Utilizing PRS for early identification also offers valuable patient education and counseling opportunities. De Mol *et al.* suggested that high-risk individuals can learn about their genetic vulnerability to MS, the implications of this susceptibility, and the importance of vigilant monitoring for early signs and symptoms [221-223].

In summary, utilizing PRS in the context of MS promises substantial impacts on disease management and patient outcomes. Using genetic data to identify high-risk individuals early enables prompt interventions, personalized monitoring, and optimized treatment strategies. However, it is essential to remember that PRS is just one aspect of risk assessment. A comprehensive understanding of an individual's total MS risk requires clinical examinations and other relevant factors alongside PRS.

#### 4.2.3 Risk Stratification

PRS have been demonstrated to be a significant and valuable tool for categorizing individuals based on their genetic predisposition to MS [203, 206]. The risk categorization discussed herein carries several implications for resource allocation and strategy development in MS management. PRS enables the identification of individuals with an increased genetic predisposition to neurodegenerative disorders [223]. Comparative analysis of an individual's PRS against a predetermined threshold or reference population facilitates categorizing individuals into distinct risk groups, including low, moderate, and high risk. Choi *et al.* asserted that this approach aids in implementing targeted interventions and allocating resources to those at higher risk of developing MS [199].

The application of PRS for risk stratification can influence the frequency and severity of clinical follow-up procedures. As Qassim *et al.* noted, individuals with a high PRS, indicating a heightened genetic predisposition, may benefit from increased monitoring and clinical surveillance [224]. Furthermore, Shams *et al.* suggested that a higher frequency of clinical visits, imaging tests, and other diagnostic procedures is necessary for individuals with elevated hereditary risks [207]. Risk classification through PRS enables focused counseling and educational interventions. Individuals with a greater genetic predisposition receive information about their increased vulnerability to MS, empowering them to participate in healthcare decision-making actively, understand their risk factors, and adhere to recommended therapies and lifestyle modifications [207, 225].

In summary, PRS facilitates categorizing individuals into various MS risk levels. This stratification process allows for resource and intervention allocation based on priority, including increased clinical follow-ups, lifestyle modifications, and early implementation of disease-modifying medications. Risk stratification through PRS can enhance patient outcomes and optimize MS management by tailoring interventions to an individual's genetic predisposition.

#### 4.2.4 Population Screening

In the context of population screening, the term 'systematic identification' refers to identifying individuals within a specific group who may be highly susceptible to a particular ailment or disease [226]. Strategies for population screening that use PRS effectively detect individuals at risk of developing conditions, such as MS [204]. Early identification allows for more efficient allocation of healthcare resources, focusing on targeted screening programs and interventions for those at the highest risk [226]. For instance, employing PRS to identify individuals with a higher predisposition for MS enables targeted assessments, diagnostic examinations, and preventative measures, proving more cost-effective and resource-efficient than comprehensive screening across the entire population. This method enhances healthcare resource allocation and the efficacy of screening initiatives [204, 207].

Given these findings, conducting further clinical assessments and considering additional variables are essential before making conclusive statements about an individual's well-being. Integrating Patient-reported Outcomes (PROs) into population screening programs for illnesses, like MS, offers a more focused and streamlined approach to identifying individuals with elevated risk profiles. Leveraging genetic information can lead to more efficient distribution of healthcare resources, potentially resulting in earlier interventions and improved health outcomes for those in greatest need.

#### 4.2.5 Clinical Trial Design

According to Slunecka *et al.,* researchers can enhance the composition of their study cohorts by including individuals with elevated genetic risks, which is achieved by incorporating genetic risk information from PRS. This approach improves the ability to identify treatment effects and assess interventions specifically suited to high-risk subgroups [208]. By combining these demographic cohorts into clinical trials, the composition of the study sample becomes more heterogeneous, encompassing individuals with higher susceptibility to the condition under investigation. Nair suggested that this enrichment method can increase the likelihood of identifying significant treatment advantages or assessing interventions designed for high-risk populations [227].

The utilization of PROs in the design of clinical trials contributes to the advancement of targeted therapeutic interventions. By identifying individuals with heightened genetic susceptibility to MS, researchers can investigate medications targeting genetic factors associated with the disease [228]. Strianese *et al.* noted that implementing a personalized strategy can result in developing more efficacious medicines that address the genetic predispositions of high-risk individuals. This technique is also valuable in evaluating therapy effectiveness and forecasting individual outcomes within clinical trials [229]. Examining potential differential treatment responses between individuals with varying genetic risk profiles can be facilitated by the genetic risk information provided by PRS [204].

PRS is also beneficial in estimating sample sizes for clinical trials, as discussed by Bader *et al.* [230]. Considering the genetic risk of the target population enables researchers to determine the necessary sample size for detecting treatment effects or assessing interventions within specific genetic risk groupings [226]. Integrating PROs into the design of clinical trials for MS and other illnesses facilitates a more precise and streamlined methodology. Genetic risk information allows researchers to optimize study populations, conduct therapy testing within specified subgroups, and potentially develop personalized treatments [204]. Based on these key findings, it can be inferred that using PRS in clinical trial design is one facet and should be integrated with other relevant components to establish robust and comprehensive research protocols.

#### 4.2.6 Counselling and Patient Education

PROs significantly impact genetic counseling and patient education, particularly concerning diseases, like MS [225]. By providing information regarding their elevated PRS, individuals can enhance their awareness of increased susceptibility to specific health conditions and the potential ramifications for their well-being. This heightened level of consciousness can catalyze individuals to engage in proactive measures to prevent, detect, and manage diseases [193]. According to Zeinomar and Chung, utilizing PROs might facilitate making well-informed decisions about lifestyle choices and disease management [231].

Alfredsson and Olsson suggested that individuals at an elevated risk of acquiring MS can receive guidance regarding environmental and lifestyle factors that could impact the progression of the disease. They may receive advice on cultivating positive behaviors, such as adhering to a well-rounded dietary regimen, engaging in regular physical activity, effectively managing stress levels, and abstaining from recognized risk factors, like smoking [33]. According to Rippe, adopting lifestyle modifications based on informed decisions can reduce the occurrence of symptoms associated with MS or delay their onset [231, 232]. It is crucial to consider an individual's genetic susceptibility to MS when making informed choices about family planning, especially for those with a high PRS. They can be informed about the potential transmission of the genetic predisposition to their offspring [207]. Providing this information empowers individuals to make informed decisions about family planning, such as pursuing genetic counseling for their partners or exploring options, like Pre-implantation Genetic Diagnosis (PGD) with assisted reproductive techniques [233]. Discovering an individual's increased genetic susceptibility to an illness, like MS, could potentially pose emotional challenges [60]. Implementing routine monitoring and timely detection strategies becomes advantageous for individuals with a high PRS. According to Qassim *et al.,* genetic counseling can guide clients regarding the appropriate frequency of check-ups, screenings, and diagnostic testing based on their genetic risk profile [224]. In genetic counseling and patient education, PRS is a valuable tool that contributes to various aspects, including increasing awareness, facilitating informed decision-making, supporting family planning, providing emotional assistance, and leading follow-up monitoring strategies.

#### 4.2.7 Precision Medicine

The application of precision medicine in the context of MS holds the potential to enhance treatment efficacy. An understanding of the biochemical foundations underlying the diverse range of neurological symptoms and variations in disease severity observed in individuals with MS is now in its nascent stages. Categorizing diseases into subgroups based on their biological attributes, rather than solely relying on clinical characteristics, can elucidate disease progression and therapy effectiveness within the field of precision medicine. Several biomarkers associated with therapeutic response are now being developed, and the field of MS treatment has already seen the licensing of over 18 disease-modifying medications [234].

Precision medicine is advanced by using PRS, which tailors therapies and treatments to an individual's genetic risk profile [235]. This field aims to deliver personalized healthcare interventions considering an individual's distinct attributes, such as genetic information [236]. The utilization of PRS can aid healthcare professionals in identifying the most effective treatment approaches by considering an individual's genetic risk profile. By examining a patient's PRS, healthcare professionals can discern individuals who exhibit an elevated susceptibility to specific medical disorders, such as MS. Healthcare providers can customize treatment approaches to better address the unique needs of these patients. This process may entail carefully selecting drugs, therapies, or interventions with superior effectiveness or safety profiles specifically for persons with elevated genetic susceptibility. Healthcare workers commonly employ PRS to identify individuals who may require heightened surveillance, prompt interventions, or specific preventive measures [208]. Shams *et al.* suggested that individuals exhibiting a higher PRS for MS may benefit from increased monitoring and prompt introduction of disease-modifying treatments. These interventions aim to mitigate disease progression and improve long-term prognoses. Precision medicine utilizes PRS to provide valuable insights for the development and execution of clinical trials [207]. By integrating PRS as a criterion for participant selection, researchers can form study cohorts consisting of individuals with a higher genetic predisposition. This enrichment enables the discernment of treatment outcomes within certain risk groupings, thus promoting the advancement of personalized medicines or interventions [235].

Proactive risk management is enhanced by implementing the Person-Environment Fit (PEF) model. This model identifies individuals who may benefit from timely interventions or preventive actions. For example, individuals with a higher PRS for cardiovascular diseases can receive advice on lifestyle modifications. These include adopting a nutritious diet, increasing physical activity, and reducing stress. Such counseling mitigates their genetic predisposition and comprehensively improves their cardiovascular health [237]. Lewis and Vassos emphasized the importance of including PRS in health risk assessments, as it provides valuable genetic risk information. Therefore, integrating PRS with other clinical and environmental factors is vital for comprehensively understanding an individual's health risks.

Moreover, integrating genetic counseling and informed consent within the precision medicine framework is crucial [204]. This integration ensures that individuals fully understand the implications and limitations of genetic risk information. Informed individuals can thus make well-considered healthcare decisions [238].

In summary, PRS significantly contributes to advancing precision medicine. It guides tailored treatment strategies, enhances disease management, facilitates risk stratification, informs clinical trial design, and enables proactive risk management. Precision medicine aims to improve patient outcomes and optimize healthcare interventions' efficacy by incorporating genetic risk information. While PRS is not a definitive diagnostic tool, it is an additional risk assessment instrument, integrating genetic data. Environmental and non-genetic factors significantly influence the development of MS. Consequently, it is essential to interpret PRS alongside clinical assessments and other relevant risk factors to thoroughly understand an individual's overall susceptibility to MS.

### 4.3 Association of PRS with Phenotypes in Neuroimaging

The present investigation involved the examination of calibrated volumetric measures of different brain regions, encompassing the entire brain, white matter, peripheral grey matter, CSF, as well as three subdivisions within the deep grey matter (namely, the thalamus, caudate, and putamen). The study centered around a sample of 467 individuals from the UCSF-EPIC cohort, who were subject to annual follow-ups over 10 years. The parameters above were examined as prospective indicators of disease development in MS (Table **2**) [207, 239].

## 5 CHALLENGES AND FUTURE DIRECTIONS

### 5.1 Identifying Causal Variants and Understanding their Functional Implications

A key challenge is understanding the functional consequences of identified variations. When a likely causal mutation is identified, researchers must ascertain its effects on gene expression, protein function, and other molecular mechanisms involved in MS pathogenesis. This requires integrating both experimental and computational approaches [240]. Muhammad *et al.* suggested that functional genomics techniques, such as chromatin immunoprecipitation sequencing and RNA sequencing, facilitate the identification of regulatory elements and gene expression patterns associated with the variant in question [241, 242].

The potential for identifying causative variations in MS is expected to strengthen with advancements in genomic technologies. These include improved sequencing techniques and the inclusion of diverse populations in larger-scale research studies [243]. Manzoni *et al.* highlighted integrating genomic data with other omics data, such as epigenomics and transcriptomics, as crucial for understanding the functional implications of polymorphisms in physiological processes [244]. Additionally, employing machine learning and artificial intelligence algorithms in analyzing complex genomic and molecular data shows promise for elucidating the complex genetic architecture of MS [245].

Understanding the functional consequences of causal variations could facilitate the development of personalized therapeutic approaches for individuals with MS. Clinicians could personalize medications by identifying distinct genetic variations associated with therapy response or disease development. This approach improves effectiveness while reducing adverse effects. In a broader context, identifying causal variations and understanding their functional consequences are critical milestones in unraveling the complex mechanisms underlying MS. Ongoing research in this field can deepen our understanding of the disease and also aid in developing more targeted and individualized therapeutic interventions for those diagnosed with MS.

### 5.2 Investigating Gene-Gene and Gene-Environment Interactions in MS Susceptibility

Gene-gene interactions, known as epistasis, relate to how one gene influences a particular trait or condition depending on the presence or absence of another gene [246]. In studying MS susceptibility, researchers have explored the interaction among genetic variants across multiple genes to determine their combined effect on an individual's risk of developing the disease [11, 247]. Various methodologies, including GWAS, candidate gene studies, and pathway-based analyses [248], have been employed to investigate this phenomenon. Identifying specific gene-gene interactions associated with MS, as Patsopoulos (2018) highlighted, may provide deeper insight into the disease's underlying molecular mechanisms and potentially reveal new therapeutic targets [11]. In MS, multiple gene-gene interactions have been observed. Notably, DDX39B has been found to activate IL7R exon 6 splicing, while simultaneously repressing sIL7R significantly. Also, increased c-Jun levels have been linked to an enhanced myelinating capacity in Fbxw7 [75, 249].

Another established genetic relationship involves the increased epistasis susceptibility among the HLA-DRB1, HLA-DQA1, and HLA-DQB1 loci. Lincoln *et al.'s* work underscored the importance of studying epistasis to fully understand MS's genetic underpinnings [52]. Data analysis revealed 25 gene pairs exhibiting epistasis due to the expression of Quantitative Trait Loci (eQTLs), while four pairs showed epistasis resulting from missense variations. Two links were previously identified in the literature: one involving NF-ϏB in regulating IP10 transcription and the other involving the direct interaction between GLI-I and SUFU, essential for generating oligodendrocyte precursor cells [250].

Gene-environment interactions refer to the complex interplay between genetic and environmental factors in determining disease susceptibility. Several environmental factors, such as low vitamin D levels, smoking, Epstein-Barr virus infection, and limited sunlight exposure, have been associated with a higher risk of developing MS [60]. Most research on gene-environment interactions in MS has focused on the interaction between HLA alleles and environmental risk factors. Studies by Olsson *et al.* and Hedstrom *et al.* suggest that the presence of high-risk HLA haplotypes, especially those containing DRB1*15:01 and lacking A*02:01, may intensify the negative correlations among childhood obesity, smoking, infectious mononucleosis, solvent exposure, and the likelihood of developing MS [251, 252]. Investigating gene-gene and gene-environment interactions in MS susceptibility often requires significant collaborative efforts, given the need to analyze genetic data from large cohorts of individuals with and without MS. Additionally, a thorough assessment of environmental factors is crucial [253, 254]. The compelling data indicate that the interaction between genetic and environmental factors significantly influences the development and progression of neurodegenerative conditions, like MS. Further research is essential to gain a more comprehensive understanding of the cellular and molecular processes involved in disease development due to these interactions.

#### 5.2.1 Integrating Genetic Data with Other Omics Approaches for a Comprehensive Understanding of MS Etiology

Integrating genetic data with other omics methods offers a more thorough understanding of MS mechanisms and etiology. Genetic investigations have identified numerous variations linked to an increased susceptibility to MS. Patsopoulos noted that GWAS and other genetic sequencing methodologies are commonly used to detect these variations. By analyzing genetic data from MS patients and comparing it to data from healthy individuals, researchers can identify specific genetic variations prevalent in those diagnosed with MS [11]. These variations are often found in genes that modulate the immune system, such as HLA genes [255]. While genetic variations associated with MS provide insights into disease likelihood, they do not fully explain its causes. Integrating genetic data with various omics techniques allows for exploring interactions between genetic factors and biological processes [256].

Transcriptomics, an essential omics technique, analyzes gene expression patterns across tissues or cell types [257]. Comparing gene expression profiles between individuals with MS and healthy individuals aids in identifying dysregulated genes and pathways. Integrating transcriptomic and genetic data can reveal specific genes or pathways affected by MS-associated genetic polymorphisms [258].

Epigenomics, another omics method, studies changes in DNA and histones that can affect gene expression while preserving the DNA sequence [259]. Ho *et al.* suggested that integrating epigenomic, genetic, and transcriptomic data offers a comprehensive view of how genetic variations and environmental factors interact to modulate gene expression and contribute to MS pathogenesis [260]. Additionally, incorporating proteomics, metabolomics, and microbiomics with genetic data provides a greater understanding of MS causes [261]. Proteomics investigates proteins in cells or tissues, metabolomics examines small molecules in biological processes [262], and microbiomics studies the microbiome's composition and function, which is linked to MS. Integrating data from these diverse omics approaches with genetic information can help identify specific proteins, metabolites, or microbial species associated with MS and clarify the involved molecular pathways [263].

In summary, integrating genetic data with transcriptomics, epigenomics, proteomics, metabolomics, and microbiomics can enhance our understanding of MS etiology. This approach allows for examining the interactions among genetic factors, gene expression, epigenetic modifications, protein compositions, metabolite concentrations, and the microbiome, aiming to deepen our understanding of the mechanisms driving MS development and progression.

## 6 TRANSLATING GENETIC FINDINGS INTO CLINICAL PRACTICE

### 6.1 Implications of Genetic Findings in MS Diagnosis and Prognosis

The genetic findings in MS have significant implications for diagnosing and predicting the disease's progression. MS, a complex condition influenced by genetic and environmental factors, has been linked to certain genetic abnormalities that increase susceptibility to MS [11]. Genetic variants in the HLA region have highlighted the immune system's role in MS. HLA-DRB1*15:01 is substantially linked to an elevated risk of MS. In contrast, non-HLA genes (including TNFRSF1A, IL7R, and IL2RA) have been connected to immunological regulation and T-cell activation. This implies that MS is essentially an immune-mediated illness [117]. More than 200 non-HLA genetic variations have been associated with MS susceptibility using GWAS. These results point to intricate genetic interactions, including dementia, demyelination, and immune responses [247]. These findings can improve diagnosis and prognosis for individuals with MS through various means.

#### 6.1.1 Differential Diagnosis

MS shares clinical characteristics with other neurological illnesses with respect to differential diagnosis, thus complicating accurate diagnosis, especially in the early stages [264]. Genetic data can be valuable in this process, providing additional evidence to support MS diagnosis. For example, genetic variants in the HLA gene region, such as HLA-DRB1*15:01, increase suspicion during diagnosis and help distinguish MS from similar diseases [11]. While not exclusive to MS, these genetic markers are crucial in differentiating it from diseases with similar clinical features [265]. Kale noted that ocular neuritis, a common initial MS symptom, might indicate MS when HLA-DRB1*15:01 is present. However, genetic findings alone are insufficient for an MS diagnosis [266]. Clinical evaluation remains essential, including medical history, physical exams, neuroimaging-like MRI scans, and other diagnostic criteria [267]. Genetic findings complement clinical data during differential diagnosis, strengthening MS suspicion [268].

Therefore, genetic findings, particularly those related to MS, can reinforce its diagnosis and distinguish it from other neurological conditions with similar symptoms. Specific genetic variants, like those in the HLA gene region, provide significant evidence for differential diagnosis. However, considering clinical manifestations and diagnostic criteria, a comprehensive assessment is necessary for an accurate MS diagnosis.

#### 6.1.2 Prognostic Markers

Regarding prognostic markers, certain genetic variations serve as indicators, shedding light on MS progression and severity. Paul *et al.* found a link between various genetic polymorphisms and severe MS or increased susceptibility to specific symptoms, like early onset or frequent relapses [269, 270]. They identified these markers to aid in predicting disease course and therapy decision-making. However, it is important to note that genetic markers are just one part of the puzzle. MS progression is also influenced by environmental factors and individual differences [271].

#### 6.1.3 Personalized Treatment Approaches

Genetic discoveries offer critical insights for devising personalized treatment strategies for MS. Pardo and Jones identified a correlation between genetic variations and differential responses to distinct Disease-modifying Therapies (DMTs) [272]. Specific genetic polymorphisms indicate increased responsiveness to certain DMTs, while others may correlate with a higher likelihood of adverse effects or treatment resistance [273]. More accurate and early diagnosis of MS may be possible with personalized techniques, such as the creation of multivariate predictive diagnostic and prognostic models that take environmental exposures and biomarkers into account [274]. These strategies also provide the chance to create and enhance techniques for creating an MS “risk score” [215]. Moreover, people with MS may have less ambiguity during their illness if reliable MS indicators of therapy response and disease progression are found and can be tracked by non-invasive equipment.

Personalization must be viewed as a dynamic interaction among persons with MS, their health care professionals, and the wider clinical system supporting them. Individuals with MS have the potential to embrace personalized therapy, as people with MS are frequent smartphone users and see smartphones as helpful and convenient in general [275]. Personalized medicine can probably put current medical practices to the test, which will impact clinician expertise and people with MS experiences navigating a complicated healthcare system. Clinical professionals will inevitably face significant obstacles in the form of expanding and intricate datasets pertaining to biological factors, environmental exposures, and lifestyle choices made by their patients [274]. Therefore, in addition to patients, doctors and health services must be involved in the planning, performing, and assessment of personalized medicine research and its application in order to guarantee that it offers value to everybody. A truly patient-centered era of personalized medicine would require such solutions to enable more seamless treatment in light of the dynamics and information of health practices that are becoming increasingly complicated. By incorporating an individual's genetic profile, healthcare providers can tailor treatment options, thereby improving therapy selection and treatment outcomes.

#### 6.1.4 Research and Drug Development

Genomic studies in MS research can significantly enhance the accuracy and specificity of identifying fundamental mechanisms underlying the disease's development and progression. Patsopoulos noted that these findings greatly augment our understanding of the molecular mechanisms and processes involved in MS development and progression [11]. According to Dara *et al.,* such data are invaluable for guiding future research and advancing drug development. They enable the identification of novel therapeutic targets and the creation of more precise and effective treatment modalities [276]. It is essential to recognize the importance of genetic discoveries while considering them in conjunction with other clinical and environmental factors for MS diagnosis and prognosis [60]. Integrating genetic information with different clinical and 'omics' data can refine diagnostic, prognostic, and personalized therapeutic approaches in MS management.

### 6.2 Multidisciplinary Approach for Integrating Genetics into Clinical Care

Incorporating genetics into clinical practice is a fundamental component of contemporary medicine. It enables healthcare practitioners to deliver customized and precise interventions informed by an individual's genetic composition [233]. The successful integration of genetics into clinical practice necessitates a multidisciplinary strategy.

#### 6.2.1 Geneticists and Genomic Specialists or Counselors

Geneticists and experts in genomics play a pivotal role in genetic testing, interpretation, and counseling of genetic information. According to Malgorzata *et al.,* these professionals assist in identifying genetic variants and mutations linked to a patient's ailment and recommend appropriate genetic tests. They are vital in understanding genetic information implications and effectively communicating these results to patients and other healthcare professionals [277].

#### 6.2.2 Genetic Counselors

Genetic counselors are highly skilled professionals who guide and support individuals and families in understanding the genetic aspects of their health-related issues. They offer comprehensive assistance throughout the genetic testing process, interpreting test outcomes and providing information about various medical treatments' potential risks and benefits. Genetic counselors or other healthcare professionals collect personal and familial health histories to ascertain potential genetic disorders. Based on this information, they help decide the appropriateness of genetic testing for individuals or their relatives [278].

#### 6.2.3 Clinicians (Physicians and Nurse Practitioners)

Integrating genetic information into the entire patient care plan requires clinical expertise. Healthcare providers must understand genetic test results comprehensively, interpret their significance accurately, and make informed decisions about treatment options, as emphasized by Malgorzata *et al.* [277]. Additionally, they must convey genetic findings to patients clearly and sensitively.

#### 6.2.4 Bioinformaticians and Data Analysts

Bioinformatics researchers conduct scientific investigations with extensive molecular datasets, including DNA, microarrays, and proteomics data. The substantial volume of data generated by genetic testing underscores the pivotal role of bioinformaticians and data analysts in processing and analyzing genomic data. Hassan *et al.* utilized advanced computer techniques to identify and examine significant genetic variants and evaluate their possible clinical implications [279, 280]. Their findings suggested that including bioinformaticians and data analysts can prove to be promising in discovering novel insights regarding disease progression in MS.

#### 6.2.5 Pharmacists

Pharmacists play a crucial role in interdisciplinary teams due to the potential impact of genetics on medication responses and metabolic processes. Crews *et al.* noted that pharmacists can use a patient's genetic profile to determine the most appropriate medication dosage and selection. This approach can enhance treatment effectiveness while minimizing adverse effects [281]. Pharmacists are significant in advancing personalized medicine, particularly in examining the influence of genetics on pharmaceutical responses and metabolic processes, known as pharmacogenomics [282]. Genetic variants can influence the body's metabolism, absorption, and utilization of drugs, leading to variations in therapeutic efficacy and the risk of side effects [283]. Therefore, pharmacists are pivotal in drug discovery and tailoring pharmacological therapy to specific patients by assessing their genetic profiles, ensuring the most effective and safe treatment.

#### 6.2.6 Psychosocial and Behavioral Specialists

Disclosing genetic information can have significant psychological and emotional ramifications for patients and their families. Oliveri *et al.* highlighted that specialists in psychological and behavioural sciences can provide crucial assistance and guidance to patients in coping with the psychosocial implications arising from genetic testing outcomes. These specialists play a vital role in supporting patients and their families during genetic investigations [284, 285]. The impact of genetic information is far-reaching, contributing valuable insights into health-related issues and potential therapeutic interventions and identifying hereditary illnesses that affect individuals and their relatives [286].

The receipt of genetic testing results can elicit a wide range of emotional and psychological responses. Oliveri *et al.* noted that some patients may feel relief upon learning that they are not susceptible to certain disorders. However, others may experience anxiety, fear, or despair upon discovering potential health issues or inherited conditions [284, 285]. Additionally, individuals might feel shame or a sense of responsibility if they carry a hereditary anomaly that could be passed on to their offspring [287]. These experts are qualified to provide appropriate counselling and support in challenging times. Healthcare professionals can aid patients and their families by helping them understand test results, addressing emotional responses, and developing effective coping strategies. A study stated that these experts can guide families in having difficult conversations about genetic information, facilitating open communication [288].

Incorporating psychosocial and behavioural specialists in genetic testing is crucial for comprehensive care that addresses the broader implications of genetic information for individuals' physical and emotional well-being and their family relationships. Healthcare professionals' expertise and practical knowledge can help individuals manage the psychological aspects of genetic testing outcomes. This support can foster resilience and enable informed healthcare decisions.

#### 6.2.7 Health Information Technology (IT) Experts

The complexity of genetic data necessitates secure storage, maintenance, and integration within the Electronic Health Record (EHR) system. Ayatollahi *et al.* asserted that health information technology specialists can ensure that genetic information is accessible, accurate, and confidential [289].

Health information technology professionals are essential in managing and securely integrating genetic data within the EHR system. The complexity of this data, which includes sensitive information about hereditary traits, potential health risks, and various genomic attributes, requires utmost caution in its storage, management, and integration to ensure accessibility, accuracy, and security [289, 290]. Given these considerations, the involvement of these experts is critical in facilitating access to appropriate genetic data for patients with MS, thus aiding in their diagnosis and treatment.

## CONCLUSION

The present investigation has examined the polygenic characteristics of genetic variations and their potential consequences for the risk of MS. In recent years, notable progress has been made in studying genetic factors associated with MS susceptibility. Accumulating evidence suggests that a complex interplay of various genetic variants influences the development of this debilitating neurological disorder. Each of these variants exerts minor individual effects, but collectively, they contribute to the overall risk. Findings from GWAS and meta-analyses have revealed a broad range of genetic loci associated with MS susceptibility. Each locus influences the disease's initiation, progression, and severity. The presence of these genetic variants, which include genes related to immune regulation, inflammation, and Central Nervous System (CNS) functions, highlights the intricate nature of MS and its complex pathophysiology.

Moreover, this analysis has emphasized the importance of integrating environmental factors with genetic susceptibility to better understand the overall risk profile associated with MS. The complexity of MS is further underscored by the potential influence of gene-environment interactions in predicting an individual's susceptibility to the disease.

The challenge of risk prediction using solely genetic information remains due to the limited impact of individual genetic differences, as indicated by their modest effect sizes. However, recent advancements in PRS, combined with clinical and environmental information, hold significant potential for enhancing risk evaluation and the implementation of personalized approaches to disease management. This review has also highlighted the need for further research to elucidate the functional implications of the identified genetic variants associated with MS. Understanding the molecular mechanisms underlying these associations could pave the way for targeted therapeutic interventions and innovative treatment modalities, ultimately improving the quality of life for individuals affected by MS.

## Figures and Tables

**Table 1:** Genetic variants and their possible mechanism of association with MS progression.

**Sr. No.**	**Gene**	**Genetic Variants (SNPs)**	**Putative Mechanism** **Involved**	**Types of MS**	**References**
1.	***HLA-DRB1***	HLA-DRB115:01	↑ Influencing specific antigens presentation to immune cells and inappropriate immune response against self-antigens ↑Inflamation and injury to CNS	RRMS	[48]
2.	***DYSF–ZNF638***	rs10191329	↓Median time to needing a walking aid; a median of 3.7 years (in homozygous carriers) ↑Brainstem abnormalities and cortical pathology in the brain tissue	Relapsing form of MS	[30]
3.	***IL-2RA***	rs2104286 (intronic)	Changes in the ratio of soluble to membrane-bound molecules ↑sIL2RA and CD25 expression on CD4^+^ T cells in MS	RRMS	[70, 195]
4.	***IL7R***	T2441 rs6897932 (exon-6)	↑ Exon 6 skipping ↑mRNA fraction and changes in the soluble/membrane-bound ratio ↑Serum IL7R	RRMS	[75, 84]
5.	***TNFRSF1A***	P46L (rs1800693) (intron-6) R92 Q (rs4149584) (exon-4)	↑Lacking exon-6, altering the soluble/membrane-bound ratio; ↑> TNFR1 ↓Frequency variants in the sporadic case, altering the contact region between TNF-α and its receptor ↑Electrostatic interactions and ligand interaction alter the receptor's signalling pathway	RRMS Relapsing MS	[86, 89, 196]
6.	***CD40***	rs1883832 T	CD40-CD40L interaction immune checkpoint ↑Both innate and adaptive immune response and inflammatory response	RRMS	[118, 197]
7.	***CD58***	rs2300747 (intronic)	↑Expression of CD58 in mononuclear cells of CIS and RRMS patients	RRMS and CIS	[93]
8.	***CLEC16A***	rs12708716	↑ Levels of two distinct CLEC16A transcripts in the thymus (not in blood); splicing regulation may be cell- or thymus-specific	Relapsing MS	[138]
9.	***IRF8***	rs17445836G (intronic)	The variant is linked to: ↓Serum type-I IFN levels ↑ IRF8 expression	SPMS	[145]
10.	***CXCR5***	rs10892307 (intronic)	Immunophenotyping using PBMCs; f expressing CXCR5	Relapsing MS	[155]
11.	***CCR5***	CCR5- Δ32	Receptor (shortened and non-functional) ↑T cell migration to inflammatory areas	PPMS, RRMS, and SPMS	[164]
12.	***CTLA4***	−319C/T (rs5742909); +49A/G (rs231775); CT60A/G (rs3087243); Jo31G/T (rs11571302)	Altered immunological response or autoimmune disease susceptibility	RRMS, SPMS	[172]
13.	***CD226***	rs763361; rs727088	↓Expression on memory T cells in MS patients	Relapsing MS	[182]
14.	**DNM3-PIGC**	rs149097173	↑Genetic enrichment in CNS tissues	RRMS	[30]
15.	**TYK2**	rs34536443	↓Tfh cells, memory B cells, and IFNAR signalling	RRMS	[185]
16.	**SLAMF1**	rs3753381	↑Activity of SLAMF1 promotor b> twofold ↑Activity of enhancer-E	Relapsing MS	[192]
17.	**TNFAIP3**	rs10499194 (intronic)	Located in the intergenic region upstream of TNFAIP3 *In vitro* ChIP-seq data set generated by the ENCODE project indicates that it lies within a target site for several transcription factors, including JunD, BAF155, and DNase hypersensitive site	RRMS, SPMS	[178]
18.	**EV15**	rs10735781 rs6680578	Alters affinity for binding of PAX6 transcription factors	RRMS	[44]

**Note: ** The table displays data regarding various genetic variations and their respective processes that have been linked to distinct forms of MS, including Relapsing-remitting MS (RRMS), Secondary Progressive MS (SPMS), and Clinically Isolated Syndrome (CIS). The genetic variations are associated with the advancement of the disease and function by exerting influence on the numerous cellular and molecular mechanisms underlying MS pathology. These genetic variations alter the immune response, and their heightened expression disrupts the progression of neurodegenerative processes.

**Abbreviations:** SNP: Single nucleotide polymorphism, MS: Multiple sclerosis, HLA: Human leukocyte antigen, PPMS: Primary progressive MS, IL-2RA: Interleukin-2 receptor alpha, TNFRSF1A: Tumor necrosis factor receptor superfamily 1A, CD40: Cluster of differentiation 40, CD58: Lymphocyte function antigen-3 (LFA-3), CLEC16A: C-type lectin domain containing 16A, IRF8: Interferon regulatory factor 8, CXCR5: C-X-C Chemokine receptor type 5, CCR5: C-C chemokine receptor 5, CTLA4: Cytotoxic T-lymphocyte associated protein 4, SPMS: Secondary progressive MS, RRMS: Relapsing-remitting MS, PAX: Paired box protein 6.

**Table 2:** PRS and their association with the MS disease progression.

**Sr. No.**	**Variables**	**Description**	**References**
1.	PRS	- Utilization of GWAS summary data - Enables the prediction of genetic susceptibility to certain diseases	[15]
2.	Calculation steps	-Selection of single SNPs -Evaluation of their effect sizes -Application of appropriate weighting -Calculation of PRS	[199-202]
3.	Utility in assessing MS risk	-Determine an individual's genetic susceptibility to MS -Assisting in the evaluation and control of associated risks	[204]
4.	Predictive power	↑PRS score indicates ↑MS risk -No guarantee of disease development	[206]
5.	Early identification	-Aids in early MS risk identification -Enables preventive measures and lifestyle modifications.	[214]
6.	DMTs	-Early initiation of DMTs based on high PRS -Optimized therapeutic outcomes and reduced disability	[218]
7.	Clinical trial design	-Enrichment of clinical trial populations - Facilitates the study of treatment effects and guides personalized care	[208]
8.	Counselling and education	-Contributes to genetic counselling -Enables family planning decisions and informed patient education	[225]
9.	Precision medicine	-Guides personalized treatments -Risk management -Clinical trial design and personalized medicine	[234]
10.	Considerations	-Should be combined with clinical evaluation and other factors for comprehensive risk assessment	[204]

**Note: ** A concise overview of the several stages encompassed in the computation of PRS, as well as its significance in evaluating the risk of MS. Additionally, the table highlights the predictive capacity of PRS and its potential for early identification and the development of disease-modifying treatments. Moreover, the chart underscores the influence of PRS on the design of clinical trials, genetic counselling, and educational initiatives, and its role in advancing precision medicine.

**Abbreviations:** PRS: Polygenic risk score, GWAS: Genome-wide association studies, SNP: Single nucleotide polymorphism, DMT: Disease-modifying therapy, MS: Multiple sclerosis.

## LIST OF ABBREVIATIONS

APC: Antigen-presenting Cells
CCR5: C-C Chemokine Receptor type 5
CD: Crohn’s Disease
CD4: Cluster of Differentiation
CHOP: C/enhancer Binding Protein Homologous Protein
CLEC116A: C-type Lectin Domain Family
CTLA-4: Cytotoxic T Lymphocyte-associated Protein-4
CXCR5: C-X-C Motif Chemokine Receptor 5
DMT: Disease-modifying Therapy
DYSF-ZNF: Dysferlin Zinc Finger Protein 638
EBV: Epstein-Barr Virus
EHR: Electronic Health Record
eQTLs: Expression Quantitative Trait Loci
EA: Educational Attainment
ER: Endoplasmic Reticulum
GE: Genotype-Environment
GLI1: Glioma-Associated Oncogene Homologue 1
GWAS: Genome Wide-Association Study
HHV-6: Human-Herpes Virus 6
HLA: Human leucocyte Antigen
HLA-DRB1: HLA class II Histocompatibility-related Beta Chain
IFN: Interferon
Ig: Immunoglobulin
IL-2: Interleukin-2
IL-7R: Interleukin-7 Receptor
IRF8: Interferon Regulator Factor 8
JAK: Janus Kinase
LD: Linkage Disequilibrium
LFA-3: Lymphocyte Function-associated Antigen
MAG: Myelin-associated Glycoprotein
MBP: Myelin Basic Protein
MHC: Major Histocompatibility Complex
MOG: Myelin Oligodendrocyte Glycoprotein
MS: Multiple Sclerosis
NF-κB: Nuclear Factor Kappa β
NK: Natural Killer Cells
OCB: Oligoclonal Bands
PEF: Person-environment Fit
PLP1: Proteolipid Protein-1
PRS: Polygenic Risk Score
RA: Rheumatoid Arthritis
SLE: Systemic Lupus Erythematosus
SNP: Single Nucleotide Polymorphism
STAT: Signal Transducer and Activator of Transcription
SUFU: Suppression of Fused Homolog
Tfh: Follicular Helper T Cells
TNF: Tumor Necrosis Factor
TNFRSF1A: TNF Superfamily 1 A
TRAP: TNF receptor-associated Periodic Syndrome
Tregs: Regulatory T Cells
TYK2: Tyrosine Kinase 2 Protein
UC: Ulcerative Colitis
UPR: Unfolded Protein Response
US: United States
